# Femoral neck width genetic risk score is a novel independent risk factor for hip fractures

**DOI:** 10.1093/jbmr/zjae002

**Published:** 2024-01-12

**Authors:** Jonathan H Tobias, Maria Nethander, Benjamin G Faber, Sophie V Heppenstall, Raja Ebsim, Tim Cootes, Claudia Lindner, Fiona R Saunders, Jenny S Gregory, Richard M Aspden, Nicholas C Harvey, John P Kemp, Monika Frysz, Claes Ohlsson

**Affiliations:** Musculoskeletal Research Unit, Translational Health Sciences, Southmead Hospital, University of Bristol, Bristol BS10 5NB, United Kingdom; Medical Research Council Integrative Epidemiology Unit, Population Health Sciences, University of Bristol, Bristol BS8 2BN, United Kingdom; Sahlgrenska Osteoporosis Centre, Centre for Bone and Arthritis Research, Institute of Medicine, Sahlgrenska Academy at University of Gothenburg, 41345 Gothenburg, Sweden; Bioinformatics and Data Center, Sahlgrenska Academy at University of Gothenburg, 40530 Gothenburg, Sweden; Musculoskeletal Research Unit, Translational Health Sciences, Southmead Hospital, University of Bristol, Bristol BS10 5NB, United Kingdom; Medical Research Council Integrative Epidemiology Unit, Population Health Sciences, University of Bristol, Bristol BS8 2BN, United Kingdom; Musculoskeletal Research Unit, Translational Health Sciences, Southmead Hospital, University of Bristol, Bristol BS10 5NB, United Kingdom; Division of Informatics, Imaging and Data Sciences, School of Health Sciences, The University of Manchester, Manchester M13 9PT, United Kingdom; Division of Informatics, Imaging and Data Sciences, School of Health Sciences, The University of Manchester, Manchester M13 9PT, United Kingdom; Division of Informatics, Imaging and Data Sciences, School of Health Sciences, The University of Manchester, Manchester M13 9PT, United Kingdom; Centre for Arthritis and Musculoskeletal Health, University of Aberdeen, Aberdeen AB24 3FX, United Kingdom; Centre for Arthritis and Musculoskeletal Health, University of Aberdeen, Aberdeen AB24 3FX, United Kingdom; Centre for Arthritis and Musculoskeletal Health, University of Aberdeen, Aberdeen AB24 3FX, United Kingdom; Medical Research Council Lifecourse Epidemiology Centre, University of Southampton, Southampton SO16 6YD, United Kingdom; NIHR Southampton Biomedical Research Centre, University of Southampton and University Hospital Southampton NHS Foundation Trust, Southampton SO16 6YD, United Kingdom; Mater Research Institute, University of Queensland, Brisbane QLD, Australia 4102; Musculoskeletal Research Unit, Translational Health Sciences, Southmead Hospital, University of Bristol, Bristol BS10 5NB, United Kingdom; Sahlgrenska Osteoporosis Centre, Centre for Bone and Arthritis Research, Institute of Medicine, Sahlgrenska Academy at University of Gothenburg, 41345 Gothenburg, Sweden; Department of Drug Treatment, Sahlgrenska University Hospital, 41345 Gothenburg, Sweden

**Keywords:** DXA, BMD, hip geometry, genome-wide association study (GWAS)

## Abstract

Femoral neck width (FNW) derived from DXA scans may provide a useful adjunct to hip fracture prediction. Therefore, we investigated whether FNW is related to hip fracture risk independently of femoral neck bone mineral density (FN-BMD), using a genetic approach. FNW was derived from points automatically placed on the proximal femur using hip DXA scans from 38 150 individuals (mean age 63.8 yr, 48.0% males) in UK Biobank (UKB). Genome-wide association study (GWAS) identified 71 independent genome-wide significant FNW SNPs, comprising genes involved in cartilage differentiation, hedgehog, skeletal development, in contrast to SNPs identified by FN-BMD GWAS which primarily comprised runx1/Wnt signaling genes (MAGMA gene set analyses). FNW and FN-BMD SNPs were used to generate genetic instruments for multivariable Mendelian randomization. Greater genetically determined FNW increased risk of all hip fractures (odds ratio [OR] 1.53; 95% CI, 1.29–1.82 per SD increase) and femoral neck fractures (OR 1.58;1.30–1.92), but not trochanteric or forearm fractures. In contrast, greater genetically determined FN-BMD decreased fracture risk at all 4 sites. FNW and FN-BMD SNPs were also used to generate genetic risk scores (GRSs), which were examined in relation to incident hip fracture in UKB (excluding the FNW GWAS population; *n* = 338 742, 3222 cases) using a Cox proportional hazards model. FNW GRS was associated with increased risk of all incident hip fractures (HR 1.08;1.05–1.12) and femoral neck fractures (hazard ratio [HR] 1.10;1.06–1.15), but not trochanteric fractures, whereas FN-BMD GRS was associated with reduced risk of all hip fracture types. We conclude that the underlying biology regulating FNW and FN-BMD differs, and that DXA-derived FNW is causally related to hip fractures independently of FN-BMD, adding information beyond FN-BMD for hip fracture prediction. Hence, FNW derived from DXA analyses or a FNW GRS may contribute clinically useful information beyond FN-BMD for hip fracture prediction.

## Lay Summary

Femoral neck width (FNW) derived from DXA scans may provide useful information about hip fracture prediction, over and above that provided by BMD measurements. Therefore, we investigated whether FNW is related to hip fracture risk independently of BMD, using a genetic approach. FNW was derived from points automatically placed on the hip in DXA scans obtained from 38 150 individuals (mean age 63.8 yr, 48.0% males) in UK Biobank. Seventy-one distinct genetic factors were found to be associated with FNW. Individuals who were predicted by their genes to have greater FNW had a higher risk of hip but not forearm fractures. In contrast, those with greater genetically determined BMD of the femoral neck had a lower risk of both hip and forearm fractures. We conclude that the underlying biology regulating FNW and BMD of the femoral neck differs, and that FNW derived from DXA analyses may contribute clinically useful information beyond BMD for hip fracture prediction.

## Introduction

Hip fractures account for the greatest impact of osteoporosis in terms of mortality, morbidity, and health economic impact.^1^ DXA-derived BMD is widely used to evaluate hip fracture risk, for which femoral neck (FN) BMD has greater predictive value compared with measurements at other sites.^2^ X-ray attenuation, used by DXA to estimate BMD, reflects both bone density and depth of bone. Given the lack of correction for depth, DXA-derived BMD is expressed as g/cm^2^ and referred to as an “areal” bone density. Alternative methods for fully correcting BMD for bone size have been proposed. For example, recalibrating lumbar spine BMD to estimate true “volumetric” bone density by dividing lumbar spine BMC by bone area (BA) raised to the power of 1.5 (based on the assumption that a vertebra represents a cuboid) corrected ethnic differences in lumbar spine BMD due to size differences.^3^ However, the same geometric model does not apply to the hip.

Current methods for deriving BMD are well established, and there is a case for retaining these but combining with a separate measure of bone size. Since the height of the FN region of interest (ROI) on DXA scans is fixed, FN-BA solely depends on average femoral neck width (FNW). As the cross section of the FN approximates to a circle, FNW may provide a reasonable estimate of depth within the FN ROI. Hip structural analysis (HSA), developed over 25 yr ago, provides an automated means of deriving FNW from hip DXA scans, as well as other geometric indices and estimates of hip strength.^4^ Using this method, Rivadeneira et al. found that as well as a lower FN-BMD, hip fracture cases had greater FNW,^5^ consistent with the expected reciprocal relationship between FNW and “volumetric” FN-BMD. This raises the possibility that prediction of hip fracture by “areal” FN-BMD might be preferentially enhanced by the addition of FNW, through the provision of missing depth information at the FN ROI. Since HSA or equivalent software is widely available, if confirmed, such understanding could be readily applied to improve hip fracture prediction by DXA through combination of FN-BMD and FNW results.

In the present study, we examined whether FNW contributes to hip fracture prediction independently of FN-BMD, using a genetic approach. First, we aimed to perform a genome wide association study (GWAS) of FNW, derived from automated annotation of hip DXA scans obtained in 38 150 individuals in UK Biobank (UKB) from which minimum FNW could be calculated (Supplementary Figure S1). Results were then used to provide genetic instruments in Mendelian randomization (MR) analyses to determine if FNW is causally related to hip fracture risk, including multivariable MR (MVMR) to establish if any such effect is independent of FN-BMD. Finally, given the recent development of genetic risk scores (GRSs) for BMD to predict fractures,^6^ we examined whether combining a FN-BMD GRS with a FNW GRS provides greater prediction of hip fractures compared with the use of a single GRS alone.

## Materials and methods

### Study population

The UKB is a prospective cohort study which recruited 500 000 adults from the United Kingdom, aged between 37 and 73 yr at a baseline visit which took place between 2006 and 2010.^7^ The participants have undergone comprehensive genetic and physical phenotyping (see website for comprehensive catalogue of variables available http://biobank.ctsu.ox.ac.uk/crystal/). The study is overseen by the Ethics Advisory Committee and received approval from the National Information Governance Board for Health and Social Care and Northwest Multi-Centre Research Ethics Committee (11/NW/0382), all participants provided informed consent for this study. As part of the UKB extended imaging study, which commenced in 2014, a number of imaging modalities, including DXA, are being collected.^8^ Information on hip fracture was obtained from linkage to the hospital episode statistics (HES) database. All UKB participants were linked, both prospectively and retrospectively at baseline. HES records began on April 01, 1997, and end date for data capture for the present study was September 30, 2021. The maximum duration of follow-up for hip fracture from the baseline visit was 15.5 yr.

### FNW measurement

We used left hip DXA scans to train an 85-point Statistical Shape Model based machine-learning system (Supplementary Figure S1A) to outline the proximal femur and acetabulum in all available images as of April 2021.^9^ A custom Python 3.0 script was developed to calculate the minimum FNW in the FN region accessible online (Faber B. *Geometric Parameters Python 3.0 Code*. 2022. https://zenodo.org/badge/latestdoi/518486087). The pixel dimension data stored in DXA DICOM images were converted to millimeters (mm). FNW was defined as the shortest distance measured between the superior and inferior FN. The inferior side of the FN was mapped with points 6–12, and the superior side with points 32–38. A line-segment approach was used to automatically calculate the narrowest distance between these points (Supplementary Figure S1A and B). A description of this approach has been published previously.^10^

### Genetic analyses

#### Preparation and quality control of genetic data in UKB

Genotyping, imputation, and quality control were performed by UKB as previously described^7^ (see Supplementary supplementary methods).

#### Femoral neck width genome-wide association study

To test the association between genetic variants and FNW, FNW was stratified by sex and adjusted for age, genotyping chip, and the first 20 ancestry principal components. Residuals resulting from female and male analyses were standardized (mean = 0, SD = 1), and then combined into a single outcome for GWAS. We used a linear mixed model assuming an additive allelic effect implemented in BOLT-LMM (v2.3) to account for cryptic population structure and relatedness. The GWAS involved high-quality genome-wide imputed v3 genetic data (~10 million SNPs, INFO >0.3, MAF > = 1%). SNPs reaching genome-wide significance (5 × 10^-8^) were taken forward for conditional association analysis. The same methods were also used to generate a GWAS for hip axis length (HAL), derived from hip outline points as previously described.^10^

#### Genome wide complex trait conditional and joint analysis (GCTA-COJO)

To detect multiple independent association signals at each of the genome-wide significant (GWS) loci, we applied approximate conditional and joint genome-wide association analysis^11^ in conjunction with a UKB reference panel using GCTA v1.93 software. Conditionally independent variants at GWAS significance level were annotated with the closest gene using bedtools^12^ v2.3.0 and the Hg19 Gene list available from https://www.cog-genomics.org/link2/.

#### Look ups, MAGMA gene-set analysis, and Gene-set enrichment analysis in FUMA

Independent FNW signals were looked up in publicly available FN-BMD summary statistics^13^ (Tables 1A and 1B and Supplementary Figure S3A). To gain an overview of which biological pathways are involved, FNW and FN-BMD^13^ GWAS summary statistics were uploaded to FUMA web-based platform^14^ to perform gene-set analysis with MAGMA v1.06 (Table 2, Supplementary Tables S4 and Supplementary S5). Gene sets were obtained from MsigDB v7.0. A total of 15 496 gene sets, including curated gene sets (5500) and gene ontology (GO) terms (9996), were available for testing. Curated gene sets consist of 9 data resources including Kyoto encyclopedia of genes and genomes (KEGG), Reactome, and BioCarta (http://software.broadinstitute.org/gsea/msigdb/collection_details.jsp#C2 for details). GO terms consists of 3 categories, biological processes, cellular components, and molecular functions. All parameters were set as default. The output of gene-set analysis contains all genes in each significant gene set (Table 2, Supplementary Tables S4 and S5).

**Table 1A:** Conditionally independent genome wide significant variants for femur neck width (chromosomes 1–9).

						**FNW GWAS**			
**SNP**	**Position**	**Closest gene**	**Distance to gene**	**EA**	**OA**	**EAF**	**Beta**	**SE**	***P***
rs2284747	chr1:17306596	MFAP2	0	T	C	0.52	0.04	0.01	1.4E-08
rs2807365	chr1:22485467	WNT4	15 005	G	A	0.32	0.04	0.01	2.5E-08
rs143778922	chr1:51037660	FAF1	0	GT	G	0.09	0.09	0.01	4.0E-12
rs2820449	chr1:219714999	SLC30A10	143 771	G	A	0.66	0.05	0.01	7.5E-13
rs767282530	chr2:19727294	OSR1	168 880	A	AGATT	0.07	0.08	0.01	9.2E-10
rs7591141	chr2:42353031	EML4	43 460	T	C	0.23	0.05	0.01	3.3E-08
rs57121650	chr2:71944847	DYSF	30 949	T	C	0.52	0.04	0.01	3.2E-08
rs11684985	chr2:72895293	EXOC6B	0	A	G	0.13	0.06	0.01	1.1E-08
rs75495843	chr3:38051211	PLCD1	0	A	G	0.03	0.12	0.02	7.2E-10
rs17235557	chr3:56419866	ERC2	0	T	C	0.67	0.04	0.01	1.5E-08
rs7636776	chr3:99840446	CMSS1	0	C	T	0.41	0.06	0.01	2.4E-15
rs6763927	chr3:141140366	ZBTB38	0	T	A	0.44	0.04	0.01	1.2E-08
rs2707450	chr4:17942560	LCORL	0	C	T	0.26	0.05	0.01	6.0E-10
rs112728585	chr4:81743389	C4orf22	0	C	A	0.02	0.14	0.03	1.2E-08
rs6824784	chr4:82275412	RASGEF1B	72 136	G	A	0.32	0.05	0.01	8.7E-10
rs2131354	chr4:145599908	HHIP	0	A	G	0.53	0.07	0.01	1.8E-21
rs1507191	chr4:151247236	LRBA	0	C	T	0.75	0.05	0.01	6.2E-10
rs77844679	chr5:170812216	NPM1	1905	G	C	0.82	0.06	0.01	9.1E-10
rs702101	chr5:171276393	FBXW11	12 161	A	G	0.60	0.04	0.01	9.0E-09
rs31778	chr5:176522036	FGFR4	0	T	C	0.29	0.05	0.01	1.2E-11
rs806790	chr6:26218920	HIST1H2AE	1209	G	A	0.58	0.04	0.01	1.3E-08
rs112540634	chr6:34623905	C6orf106	0	T	C	0.14	0.06	0.01	1.5E-08
rs2258604	chr6:34997606	ANKS1A	0	A	T	0.66	0.05	0.01	1.8E-12
6:35038589_CCT_C	chr6:35038589	ANKS1A	0	CCT	C	0.98	0.18	0.03	9.0E-10
6:158783382_AT_A	chr6:158783382	TULP4	0	AT	A	0.33	0.05	0.01	1.7E-10
rs798565	chr7:2752152	AMZ1	0	G	A	0.70	0.05	0.01	2.9E-11
rs148066163	chr7:47169380	TNS3	145 373	CAA	C	0.28	0.04	0.01	1.6E-08
rs42039	chr7:92244422	CDK6	0	T	C	0.24	0.06	0.01	2.1E-15
rs34275932	chr7:120816329	CPED1	0	C	G	0.59	0.04	0.01	1.6E-09
rs149882987	chr7:148584494	EZH2	3081	G	A	0.03	0.14	0.02	6.3E-11
rs72656010	chr8:57122215	PLAG1	0	T	C	0.87	0.06	0.01	4.1E-10
rs75810927	chr8:69587226	C8orf34	0	C	A	0.77	0.05	0.01	1.3E-08
rs9298310	chr8:79164782	PKIA	263 593	G	C	0.29	0.07	0.01	1.5E-22
rs28705285	chr9:98279801	PTCH1	462	G	T	0.24	0.05	0.01	4.0E-09
rs762624732	chr9:99106848	SLC35D2	0	T	TA	0.20	0.05	0.01	3.4E-08
rs10123619	chr9:119353611	ASTN2	0	A	G	0.15	0.06	0.01	5.9E-10

Conditionally independent significant signals within chromosomes 1–9 associated with femur neck width (FNW) in 38 150 UKB participants. Results are presented as estimated association (beta) and standard error (SE) expressed per effect allele (EA). Beta, SE, and *P* are from the conditional (COJO) analysis.

OA, other allele; EAF, effect allele frequency.

**Table 1B:** Conditionally independent genome wide significant variants for femur neck width (chromosomes 10–21).

						**FNW GWAS**			
**SNP**	**Position**	**Closest gene**	**Distance to gene**	**EA**	**OA**	**EAF**	**Beta**	**SE**	***P***
rs10828316	chr10:22838389	PIP4K2A	0	C	A	0.33	0.05	0.01	3.2E-10
rs3740237	chr10:32557592	EPC1	0	G	C	0.86	0.06	0.01	8.1E-09
rs12776235	chr10:95019809	MYOF	46 378	T	C	0.53	0.04	0.01	6.7E-09
rs7952436	chr11:67024534	KDM2A	0	C	T	0.92	0.12	0.01	2.4E-20
rs923346	chr11:68182375	LRP5	0	T	C	0.83	0.06	0.01	1.5E-09
rs11609223	chr12:1419127	ERC1	0	G	T	0.68	0.04	0.01	8.5E-09
rs76895963	chr12:4384844	CCND2	0	G	T	0.02	0.19	0.03	2.4E-12
rs11046703	chr12:23114157	ETNK1	270 558	C	T	0.05	0.10	0.02	8.4E-11
rs59932020	chr12:24179601	SOX5	75 635	T	A	0.22	0.06	0.01	4.3E-12
rs5029277	chr12:28022055	KLHL42	66 082	G	A	0.78	0.07	0.01	5.2E-15
rs11049385	chr12:28320492	CCDC91	0	G	A	0.31	0.05	0.01	2.2E-12
rs10878984	chr12:69828534	FRS2	35 596	T	C	0.34	0.05	0.01	3.6E-11
rs7954185	chr12:94096173	CRADD	0	A	T	0.49	0.05	0.01	5.2E-14
rs71190381	chr13:51120097	DLEU1	0	G	GAGTGA	0.79	0.08	0.01	6.5E-23
rs12889267	chr14:21542766	ARHGEF40	0	A	G	0.83	0.05	0.01	3.4E-09
rs10083313	chr14:53917808	DDHD1	297 808	A	C	0.27	0.05	0.01	9.1E-10
rs28929474	chr14:94844947	SERPINA1	0	T	C	0.02	0.14	0.03	3.0E-08
rs569147467	chr14:103855865	MARK3	0	C	CA	0.33	0.04	0.01	1.4E-08
rs200675402	chr15:86894913	AGBL1	0	A	AT	0.97	0.13	0.02	2.5E-08
rs8034564	chr15:99190601	IGF1R	1600	G	A	0.42	0.04	0.01	1.0E-08
rs30224	chr16:14406119	MKL2	45 489	T	C	0.35	0.04	0.01	1.6E-09
rs7223535	chr17:29211667	ATAD5	0	G	A	0.73	0.05	0.01	2.8E-10
rs1043515	chr17:36922196	PIP4K2B	0	G	A	0.57	0.05	0.01	2.2E-14
rs9905385	chr17:59498250	C17orf82	7609	A	G	0.33	0.04	0.01	7.9E-09
rs4141079	chr17:59531402	TBX4	0	A	C	0.74	0.05	0.01	4.3E-10
rs4968440	chr17:59613258	TBX4	50 787	C	G	0.36	0.04	0.01	3.6E-08
rs17779649	chr17:70372779	SOX9	250 218	A	C	0.87	0.08	0.01	1.2E-15
rs9912553	chr17:79959703	ASPSCR1	0	G	C	0.72	0.05	0.01	5.5E-10
rs4369779	chr18:20735408	CABLES1	0	C	T	0.79	0.05	0.01	1.1E-08
rs1074047	chr19:2158748	AP3D1	0	G	A	0.48	0.05	0.01	2.9E-11
rs742630	chr20:31350664	DNMT3B	0	C	G	0.60	0.04	0.01	1.2E-08
rs149142833	chr20:32188142	CBFA2T2	0	C	T	0.84	0.06	0.01	2.3E-09
rs143384	chr20:34025756	GDF5	0	G	A	0.41	0.10	0.01	4.0E-48
rs6063031	chr20:45522102	EYA2	1162	A	G	0.45	0.05	0.01	9.0E-13
rs2298333	chr21:39673981	KCNJ15	0	C	T	0.43	0.04	0.01	1.5E-09

Conditionally independent significant signals within chromosomes 10–21 associated with femur neck width (FNW) in 38 150 UKB participants. Results are presented as estimated association (beta) and standard error (SE) expressed per effect allele (EA). Beta, SE, and *P* are from the conditional (COJO) analysis.

OA, other allele; EAF, effect allele frequency.

**Table 2:** MAGMA gene set analysis.

**SET**		**VARIABLE**	**NGENES**	***P*-value**
**FNW**				
_SET1_		Curated_gene_sets:pid_hedgehog_2pathway	22	9.5E-10
_SET2_		Curated_gene_sets:nikolsky_breast_cancer_20q11_amplicon	31	2.9E-15
_SET3_		Curated_gene_sets:reactome_ligand_receptor_interactions	8	1.7E-06
_SET4_		Curated_gene_sets:reactome_gli_proteins_bind_promoters_of_hh_responsive_genes_to_promote_transcription	7	5.5E-09
_SET5_		GO_bp:go_cartilage_development	205	2.7E-11
_SET6_		GO_bp:go_regulation_of_cartilage_development	65	4.6E-09
_SET7_		GO_bp:go_osteoblast_differentiation	203	6.4E-08
_SET8_		GO_bp:go_skeletal_system_development	513	2.4E-10
_SET9_		GO_bp:go_chondrocyte_development	47	1.0E-07
_SET10_		GO_bp:go_chondrocyte_differentiation	117	2.7E-16
_SET11_		GO_bp:go_regulation_of_chondrocyte_differentiation	47	2.9E-10
_SET12_		GO_bp:go_positive_regulation_of_epidermis_development	37	8.1E-07
_SET13_		GO_bp:go_positive_regulation_of_cartilage_development	30	4.9E-10
_SET14_		GO_bp:go_ossification	373	6.3E-08
_SET15_		GO_bp:go_regulation_of_osteoblast_differentiation	111	2.3E-06
_SET16_		GO_bp:go_connective_tissue_development	267	5.0E-08
_SET17_		GO_bp:go_positive_regulation_of_chondrocyte_differentiation	20	8.5E-09
_SET18_		GO_bp:go_animal_organ_morphogenesis	1027	2.4E-07
_SET19_		GO_mf:go_proximal_promoter_sequence_specific_dna_binding	529	2.2E-07
**FN-BMD**				
_SET1_		Curated_gene_sets:reactome_runx1_regulates_transcription_of_genes_involved_in_wnt_signaling	5	4.3E-07

Significant gene sets from the MAGMA gene-set analysis of the FNW GWAS and the FN-BMD GWAS (PMID: 26367794): SET and VARIABLE: name of the gene set, NGENES: number of genes in the gene set. In total 15 496 gene sets were available for testing; 15 488 of them were represented in the FNW GWAS and 15 485 were represented in the FN-BMD GWAS. A gene set was considered to be significant if *P* < .05/15496 = 3.2 x 10^-6^.

Based on the genes at the identified loci, we also performed a gene-set enrichment analysis as implemented by the FUMA SNP2GENE function (Supplementary Tables S6 and Supplementary S7). We also looked up the independent FNW signals in the GWAS catalogue (Supplementary Table S8).

#### Genetic correlation

To estimate the genetic correlation between FNW and related traits, we used (cross-trait) linkage disequilibrium score regression (LDSR)^15^ as implemented in the LD score tool LDSC available on github.^16^ This method uses the cross-products of summary test statistics from 2 GWASs and regresses them against a measure of how much variation each SNP tags (its LD score). The LDSR analyses were restricted to HapMap3 SNPs with minor allele frequency (MAF) > 5% in the 1000 Genomes European reference population. We used precalculated LD scores from the same reference population (https://data.broadinstitute.org/alkesgroup/LDSCORE/). We estimated the genetic correlation between FNW and 3 related traits: hip fracture,^17^ fracture at any bone site,^18^ and FN-BMD,^13^ using public available GWAS summary statistics. We accounted for multiple testing by using a conservative Bonferroni correction for 3 tests (*P* = .05/3 = .017).

#### Mendelian randomization

To assess the effects of FNW on the risk of fractures at different bone sites, we performed 2-sample Mendelian randomization (MR) analyses. We used a MVMR approach to estimate the independent causal associations for genetically determined FNW and FN-BMD with risk of fractures at different bone sites. Genetic instrument variables for the FNW exposure were derived from the present GWAS on FNW, while genetic instruments for FN-BMD were derived from previous GWAS on FN-BMD.^13^^,^^19^ The highest available number of FN-BMD signals (*n* = 49) was identified in the FN-BMD GWAS by Estrada et al.^19^ However, HapMap imputation was used in that early GWAS meta-analysis, hindering the ability to examine FN-BMD associations for FNW signals identified in the more detailed imputation panel used by UKB. We therefore selected the GWS FN-BMD signals from Estrada et al. as instruments for FN-BMD but used effect estimates from the later FN-BMD GWAS study by Zheng et al.^13^ (the imputation panel in the latter included the majority of FNW GWAS SNPs identified here, making MVMR feasible).

We only used variants that were available in both the present FNW GWAS and the FN-BMD GWAS by Zheng et al. The variants were required to have a MAF > 1% and to be associated with either FNW or FN-BMD at a GWS level (*P* < 5 × 10^-8^). We selected instruments with a pairwise *r*^2^ < 0.01 (based on the European populations in LDlink)^20^ to ensure that there was low correlation between instruments. One SNP associated with FNW was strongly correlated with one SNP associated with FN-BMD (*r*^2^ = 0.73). For this pair, we first selected the SNP that is most strongly associated with FN-BMD in the GWAS. In sensitivity analyses, we selected the other SNP that is most strongly associated with FNW, revealing virtually identical results.^21^ Two palindromic FNW SNPs with non-clear strand were removed from the analyses. After quality filtering, 40 FNW SNPs and 40 FN-BMD SNPs remained. Using Steiger filtering, no more FNW SNPs were suggested to be removed. We estimated the F-statistic as a measure of instrument strength.^22^

The outcome fracture associations (logistic regression adjusted for age, and sex) used in the 2-sample MR were derived from summary statistics from a previous GWAS on hip fractures (including 11.515 hip fracture cases^17^) or newly performed association analyses in UKB, excluding the FNW GWAS data set (Supplementary Table S1). As the primary MR analyses, we used combined weighted estimates by IVW using fixed or random effects depending on Cochran’s Q statistic test of heterogeneity. We then used weighted median MR as a sensitivity analysis and MR-Egger regression to test for possible directional horizontal pleiotropy. To reduce the possible impact of heterogeneity, we also performed sensitivity analyses excluding outliers of genetic instruments using MR-LASSO. The MR analyses were conducted using the R-package MR.

#### Weighted GRSs

We defined a weighted GRS for FNW (FNW GRS) based on the 71 conditionally independent (COJO) significant SNPs identified in the present study. We also defined a GRS for FN-BMD (FN-BMD GRS) based on 49 SNPs previously identified to be associated with FN-BMD at GWS level.^19^ For each individual, the GRSs were defined as the weighted sum of SNP dosages, where SNP effects from the corresponding BMD GWAS were used as weights. The GRSs were standardized to have a mean of and SD of 1. The separate and combined associations for FNW GRS and FN-BMD GRS with incident fracture were calculated using Cox-regression in UKB samples excluding the samples used for the FNW GWAS. The effects are given as hazard ratios (HRs) per SD increase in GRS. The base model included sex and baseline age as covariates.

## Results

### Genome-wide association study

The discovery set comprised 38 150 UKB participants with available FNW analyses, mean 63.8 yr, of whom 48% were male (Table 3). We identified 71 independent signals at 61 loci passing GWS level (*P* < 5 × 10^−8^; Tables 1A and 1B; Supplementary Table S2 and Supplementary Figure S2), of which 8 were low-frequency (MAF ≤ 5% but >1%) and 63 were common (MAF > 5%). These 71 signals explained 7.6% of the variance of FNW (Supplementary Table S2) and showed limited associations with FN-BMD as evaluated in a previous FN-BMD GWAS data set^13^ (Supplementary Table S3, Supplementary Figure S3A). Conversely, the previously identified 49 GWS FN-BMD signals showed limited associations with FNW in the present GWAS dataset (Supplementary Figure S3B).

**Table 3:** Characteristics of UKB Study participants in the FNW GWAS.

	**All**	**Males**	**Females**
	(*N* = 38 150)	(*N* = 18 314)	(*N* = 19 836)
Age (yr)	63.8 (7.5)	64.5 (7.6)	63.1 (7.4)
Weight (kg)	75.4 (15.1)	83.3 (13.4)	68.2 (12.8)
Height (cm)	170.2 (9.4)	177.3 (6.6)	163.7 (6.3)
FNW (mm)	31.7 (3.5)	34.6 (2.4)	29.0 (2.0)

Population characteristics of the UKB participants in the FNW GWAS with complete FNW and covariate data.

MAGMA gene set analyses of the FNW GWAS data identified different gene sets (representing cartilage differentiation, hedgehog signaling, skeletal development) compared with FN-BMD GWAS (runx1/Wnt signaling), demonstrating distinct underlying biology for the regulation of FNW and FN-BMD (Table 2, Supplementary Tables S4 and S5). This notion was further supported by clear differences in FUMA gene set enrichment for FNW and FN-BMD signals (Supplementary Tables S6 and S7), with the FNW GWAS comprising signals for hip dimensions, fat distribution, and height, while the FN-BMD GWAS included signals for fracture risk and BMD at other sites.

Look-up in the GWAS catalogue of the 71 conditionally independent FNW signals including linked signals (*r*^2^ > 0.8) identified multiple signals for osteoarthritis, hip circumference, and hip bone size (Supplementary Table S7). We observed a strong genetic correlation for FNW with hip fractures (r_g_ = 0.48) but not with fractures at any bone site (r_g_ = 0.07) (Supplementary Table S9). A modest inverse genetic correlation was observed for FNW with FN-BMD (r_g_ = -0.20). A second parameter of hip geometry, HAL, could be derived from our automated annotation of hip shape, for which GWAS summary statistics were also obtained. Although HAL was strongly correlated genetically with FNW (r_g_ = 0.61), this showed a considerably weaker genetic correlation with hip fracture (r_g_ = 0.24).

### Mendelian randomization

SNPs selected from the current FNW GWAS, and previous FN-BMD GWAS,^19^ provided well-powered genetic instruments for subsequent MR analyses (F-statistic 40.6 and 31.0, respectively, Supplementary Table S10). We evaluated the causal associations for genetically determined FNW and FN-BMD with hip fractures using a hip fracture GWAS meta-analysis for the outcome analyses^17^ (Supplementary Table S11 and Supplementary Figure S4). Univariate MR revealed a significant effect of both genetically determined FNW (OR = 1.55, 95% CI, 1.35–1.78, per SD increase) and genetically determined FN-BMD (OR = 0.44, 95% CI, 0.37–0.53) on hip fracture risk. Similar results were observed in MVMR including both genetically determined FNW (FNW OR = 1.35, 95% CI, 1.16–1.59) and genetically determined FN-BMD (FN-BMD OR = 0.48, 95% CI, 0.40–0.57; Supplementary Table S11 and Figure S4) as exposures.

**Figure 1:**
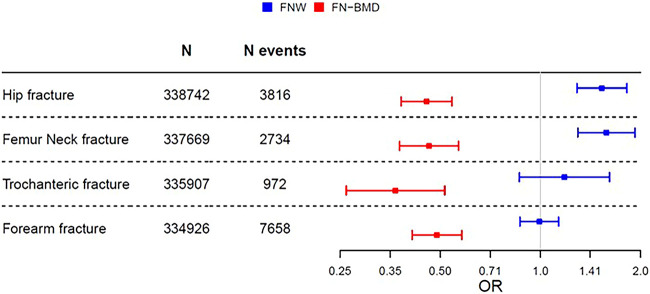
Independent effects of genetically determined FNW and FN-BMD on fracture risk in UKB: MVMR analyses to estimate the effect of genetically determined FNW and femoral neck bone mineral density (FN-BMD) on the risk of hip fracture, FN fractures, trochanteric fractures, and forearm fractures in UKB. Both prevalent and incident fractures were included. OR and 95% CIs are given.

We next evaluated the effects of genetically determined FNW and FN-BMD on fractures at different bone sites in the UKB data set excluding subjects included in the FNW GWAS (Figure 1, Supplementary Table S12, Supplementary Figures S5–S8). MVMR revealed that high genetically determined FN-BMD was causally associated with reduced risk of all hip, FN, trochanteric, and forearm fractures (Figure 1, Supplementary Table S12, Supplementary Figures S5–S8). In contrast, high genetically determined FNW was causally associated with increased risk of all hip fractures (OR = 1.53, 95% CI, 1.29-1.82) and FN fractures (OR = 1.58, 95% CI, 1.30-1.92), but not trochanteric (OR = 1.18, 95% CI, 0.86–1.61) or forearm (OR = 0.99, 95% CI, 0.88–1.11) fractures (Figure 1, Supplementary Table S12, Supplementary Figures S5–S8). Similar results were observed in univariate and multivariate MR analyses (Supplementary Table S12).

As an alternative exposure in the MR analyses, we used a weighted GRS for FNW (FNW GRS, 71 SNPs). Using this FNW GRS as exposure, MR confirmed that FNW is causally associated with all hip (OR = 1.64, 95% CI, 1.34–2.02, per SD increase) and femur neck (OR = 1.85, 95% CI, 1.45–2.36) but not trochanteric (OR = 1.22, 95% CI, 0.81–1.83) fractures. In sex stratified analyses, the causal effects of FNW on all hip fractures and FN fractures were slightly greater in women compared to men (Supplementary Table S13). Collectively, these data demonstrate that genetically determined FNW is causally associated with the risk of hip fractures, specifically that of FN fractures.

### FNW GRS and FN-BMD GRS add independent information for prediction of incident hip fractures

**Figure 2:**
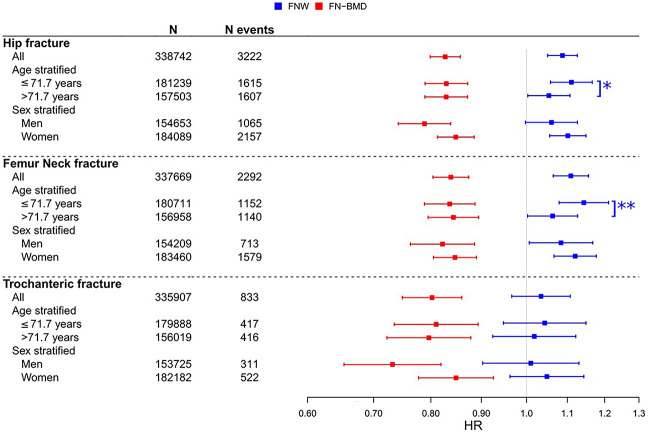
Associations for FNW GRS and FN-BMD GRS with incident fractures in UKB The effects are given as HRs per SD increase in GRS. All models were adjusted for sex and baseline age, except the sex stratified models where sex was not included as a covariate. Age stratification was based on the median age at hip fracture (71.7 yr). ^*^Significant age interaction (*P* = 1.3 × 10^-4^) for FNW GRS. ^*^^*^Significant age interaction (*P* = 1.9 × 10^-3^) for FNW GRS.

We next determined whether the FNW GRS and/or FN-BMD GRS predict incident hip fractures**.** In separate models, a high FN-BMD GRS was associated with reduced risk of incident hip fracture at any site (HR 0.83; 0.80–0.86 per SD increase), whereas a high FNW GRS was associated with increased hip fracture risk (HR 1.09; 1.05–1.13; Figure 2, Supplementary Table S14). The associations between the FNW GRS and hip fracture risk were more pronounced for FN (HR 1.11; 1.06–1.16) compared with trochanteric (HR 1.03; 0.97–1.11) fractures. Similar results were observed in combined analyses (including both FNW GRS and FN-BMD GRS) and in models additionally adjusted for BMI; however, the association between FNW GRS and hip fracture risk was attenuated by approximately 25% following separate adjustment for height and weight to account for effects of body size (Supplementary Table S14). An age interaction (*P* = 1.9 × 10^-3^ for the age/FNW GRS interaction term) was observed for the association between the FNW GRS and FN fracture risk, reflected by a higher HR for younger (age ≤ 71; 1.14; 1.08-1.21) compared with older (age > 71; 1.06; 1.00-1.13) individuals (Figure 2, Supplementary Table S15). Similar associations for the FNW GRS with incident hip fractures were observed in both men and women (Figure 2, Supplementary Table S15). Additionally, we observed an interaction between the FN-BMD GRS and the FNW GRS for the prediction of hip fracture risk (*P* = .04 for the interaction term), where individuals with genetically determined low FN-BMD had a more pronounced increased risk from genetically determined high FNW.

**Figure 3:**
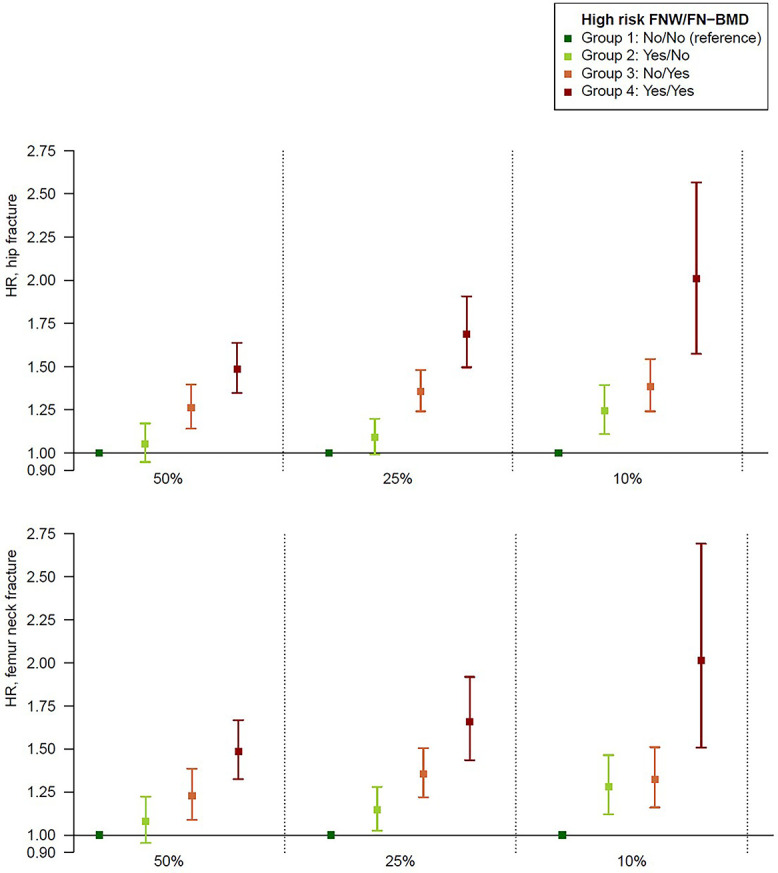
Additive associations for binarized high risk FNW GRS and, binarized high risk FN-BMD GRS with fractures risk. Participants in UKB were divided into 4 different groups (no/no, yes/no, no/yes and yes/yes) based on their binarized FNW GRS and binarized FN-BMD GRS, using 3 different cut-off limits as definitions of high risk for the 2 GRS (50%, 25%, and 10 %).

Finally, we examined additive associations for binarized high risk FNW GRS and binarized high risk FN-BMD GRS with hip fractures risk. Participants in UKB were classified as high risk (yes/no) for high FNW based on their FNW GRS and at high risk (yes/no) for low FN-BMD based on their FN-BMD GRS. Participants were divided into 4 different groups (group 1 = no/no; group 2 = yes/no; group 3 = no/yes; and group 4 = yes/yes) based on their binarized FNW GRS and binarized FN-BMD GRS. We used the lowest risk group (group 1 = no/no) as reference to study the possible additive associations for the 2 different GRSs. We used 3 different cut-off limits as definitions of high risk for the 2 GRS (50%, 25%, and 10%; Figure 3). A high binarized FNW GRS was associated with high hip fracture risk, while a low binarized FN-BMD GRS was associated with high hip fracture risk. Using the 10% cut-off for high risk, subjects in the high-risk GRS group for both FNW GRS and FN-BMD GRS (ie, group 4 = yes/yes) had a more than 2-fold increased risk of hip fractures compared with those in the low-risk group for both binarized GRSs (ie, group 1 = no/no). Binarized FNW GRS and FN-BMD GRS contributed independently to hip fracture prediction. For example, on comparing groups 3 and 4, the high risk FNW GRS added information beyond the high risk FN-BMD GRS (Figure 3, Supplementary Table S16).

## Discussion

We investigated whether FNW contributes to hip fracture risk independently of FN-BMD, using a genetic approach. Having performed a GWAS of FNW derived from hip DXA scans in over 38 000 individuals in UKB, we identified 71 conditionally independent signals in 61 different loci explaining 7.6% of the variance of FNW, of which 70 signals represented novel genetic associations with FN bone size (the FN-area signal at the HHP locus was previously reported by Styrkarsdottir et al. ^23^). The genetic architecture of FNW appeared to be distinct to that of FN-BMD, suggesting these 2 traits are in large part independent. Less than 20% of FNW genetic signals were nominally associated with FN-BMD. FNW showed a relatively weak genetic correlation with FN-BMD, and MAGMA gene set analysis revealed involvement of FNW and FN-BMD SNPs in distinct biological pathways.

Although FNW only showed weak genetic correlation with FN-BMD, it was correlated relatively strongly with hip fractures. Given the suggestion that FNW is largely independent of FN-BMD, we investigated whether FNW is causally related to the risk of hip fracture, independently of FN-BMD. MVMR revealed that greater FNW, or a highly correlated hip shape parameter, increases the risk of any hip fractures, and that of FN fractures specifically, despite adjustment for FN-BMD. In contrast to FNW which was only related to risk of hip fracture/FN fracture, FN-BMD was also related to risk of trochanteric and forearm fractures.

A GRS based on genetic associations with FN-BMD has previously been proposed as an adjunct in clinical fracture prediction, either in isolation or in combination with clinical risk factors such as those included in FRAX.^6^^,^^24^ Therefore, we examined whether a GRS based on FNW might also have utility in hip fracture prediction. We found that both FNW and FN-BMD GRSs were independently related to risk of hip/FN fracture, and that these exerted additive effects on hip fracture risk. For example, an individual with a GRS in the highest 10% risk category for both FNW and FN-BMD has an approximately 2-fold increased risk of hip/FN fracture, compared with a 25%–35% increase based on either parameter alone. In addition to improving the use of GRSs to predict fractures by combining 2 independent scores, an FNW GRS may also prove useful due to its presumed independence from DXA BMD. This would represent an important advantage over a BMD GRS, which provides little additional predictive value if BMD is already known.^25^

Though the present study focused on additive effects of GRSs for FNW and BMD on risk of hip fracture, it may be possible to use an equivalent approach based on measured parameters. In the present study, FNW was derived using a bespoke method based on points annotated as part of a separate study on hip shape.^9^ However, an equivalent measure, obtained using HSA software,^4^ is widely available (these were strongly correlated [*r*^2^ = 0.97] in a subset of 1744 DXA images where FNW was obtained using both methods). An alternative method would be to combine BMD with FN BA, which is provided routinely during hip DXA measurements, and also correlated strongly with FNW (*r*^2^ = 0.93).

When used alone, the FNW GRS had a clear independent relationship with hip fracture risk, in the opposite direction to that of FN-BMD and when combining both GRSs, a marked improvement in predictive ability was observed. One explanation for these findings is that the input of additional information about bone size, provided by FNW, enables a more accurate estimate of “volumetric” BMD than that provided by “areal” BMD, by providing missing information about depth. As discussed in the introduction, several approaches have been attempted to more fully account for bone size when evaluating BMD by DXA. Our results suggest that this concept can also be applied to GRSs used for fracture prediction. Rather than providing missing depth information, it may be that greater FNW per se has a negative effect on bone strength and fracture risk. For example, for a given cortical thickness, greater FNW is inversely related to resistance to buckling as reflected by buckling ratio.^5^ On the other hand, FNW is positively related to bending strength as reflected by cross-sectional moment of inertia^26^; to the extent that both types of forces contribute to risk of hip fracture, whether FNW has any net direct effect on hip fracture risk is currently unclear. A further explanation is that, rather than a direct association, FNW is related to hip fracture risk through co-association with height, which is also positively related to risk of hip fracture,^27^ possibly because height is a proxy for leg length.^28^

Though the overall genetic correlation between FNW and FN-BMD was relatively weak, for those SNPs related to both traits, an inverse relationship between these 2 traits was generally observed. However, the *LRP5* locus was an exception, since this was positively related to both FN-BMD and FNW, suggesting that in contrast to other loci, the *LRP5* locus has a positive effect on both bone size and BMD. In-keeping with this suggestion, individuals with high bone mass as a consequence of a mutation in *LRP5* have been found to have both increased BMD and bone size, as reflected by tibial and radial cortical area and thickness.^29^ Although the *LRP5* locus appears to affect bone size of the skeleton as a whole, genetically determined FNW only influenced risk of FN/hip fracture, suggesting fractures at the latter site are particularly influenced by bone geometry. This contrasts with the genetically determined FN-BMD GRS, which influenced fracture risk at multiple sites, suggesting that FN-BMD signals influence BMD throughout the skeleton. In addition to being restricted to the prediction of FN/hip fractures, the FNW-GRS was more strongly related to risk of hip fracture in younger individuals. One possible explanation for this finding is the greater contribution to hip fracture of risk factors unrelated to skeletal fragility in older individuals, such as factors related to fall risk.

This paper reports the first FNW GWAS, which provided the basis for a novel FNW GRS which may have clinical utility as a BMD independent risk factor for hip fracture. In terms of limitations, though many novel loci related to FNW were identified, functional genomic studies intended to characterize genetic mechanisms contributing FNW were not undertaken as part of the current study. That said, MAGMA gene set analysis was used to characterize the biological pathways identified by our GWAS. These primarily comprised genes representing cartilage differentiation, hedgehog signaling, and skeletal development, consistent with the determination of overall skeletal size. This contrasted sharply with findings for FN-BMD, which primarily identified Wnt signaling genes, consistent with the important role of Wnt signaling in the regulation of bone mass.^30^

In terms of other limitations, UKB on which this GWAS was based is primarily comprised of Caucasians, and further studies are required to investigate whether the GRS has equivalent predictive value in other ethnic groups. To establish the clinical utility of our FNW GRS, further studies are required to confirm that this GRS predicts hip fracture independently of BMD, as well as established clinical risk factors. Furthermore, it should be acknowledged that although the FNW GRS may have clinical utility for predicting fractures, unlike BMD, this appears to be limited to hip fractures. Finally, although we have described relationships between genetically predicted FNW and BMD, and hip fracture, associations between hip fracture and directly measured FNW and BMD were not presented. The latter analyses are restricted to the subgroup with DXA data, and given this smaller sample and the shorter follow-up period, there were relatively few hip fractures on which to base analyses, limiting statistical power. The number of participants undergoing DXA scans in UKB, as well as the duration of follow up, is increasing substantially with time, and we plan to reexamine these relationships once more hip fracture cases are available.

In conclusion, our FNW GWAS demonstrates that the biology underlying this trait differs from that of FN-BMD. Consequently, although FNW or a highly correlated hip shape parameter is causally related to hip fractures, this is independent of FN-BMD, and DXA-derived FNW adds information beyond FN-BMD for hip fracture prediction. Based on the genetic evidence presented herein, we propose that FNW derived from DXA analyses or a FNW GRS may add clinically useful information beyond FN-BMD for hip fracture prediction.

## Supplementary Material

Supplementary_Figures_Rev_zjae002

Supplementary_Tables_Rev_zjae002

Supplementary_Methods_zjae002

## Data Availability

The femoral neck GWAS summary statistics will be available from the GWAS catalog.
