# Supplementary material for: Femoral neck width genetic risk score is a novel independent risk factor for hip fractures
Source: J Bone Miner Res. 2024 Jan 12;39(3):241–51. doi: 10.1093/jbmr/zjae002 (PMC11240160; doi:10.1093/jbmr/zjae002)
Supplement: Supplementary_Figures_Rev_zjae002 [file Supplementary_Figures_Rev_zjae002.pptx]

## Slide 1
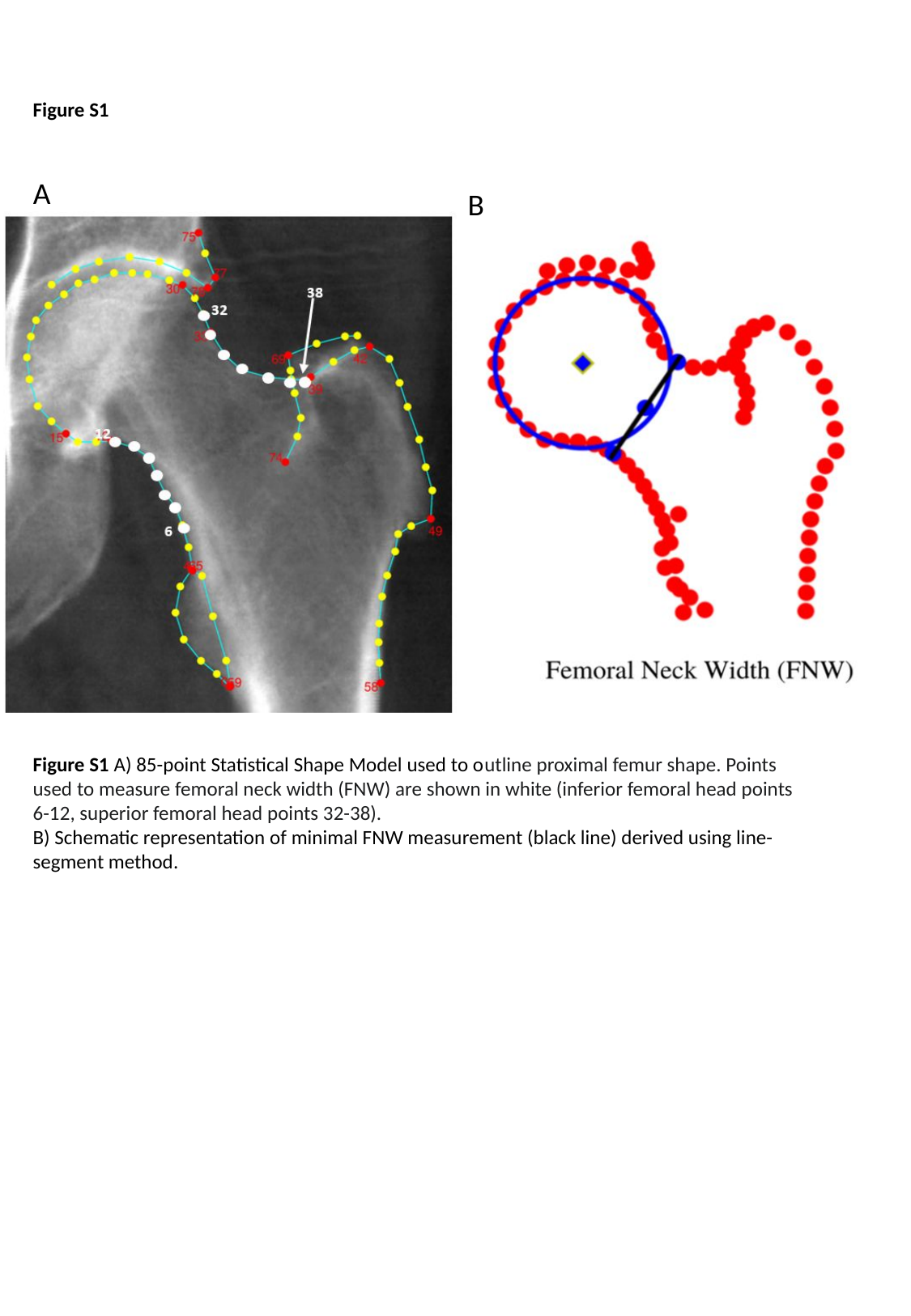

Figure S1
A
B
Figure S1 A) 85-point Statistical Shape Model used to outline proximal femur shape. Points used to measure femoral neck width (FNW) are shown in white (inferior femoral head points 6-12, superior femoral head points 32-38).
B) Schematic representation of minimal FNW measurement (black line) derived using line-segment method.

## Slide 2
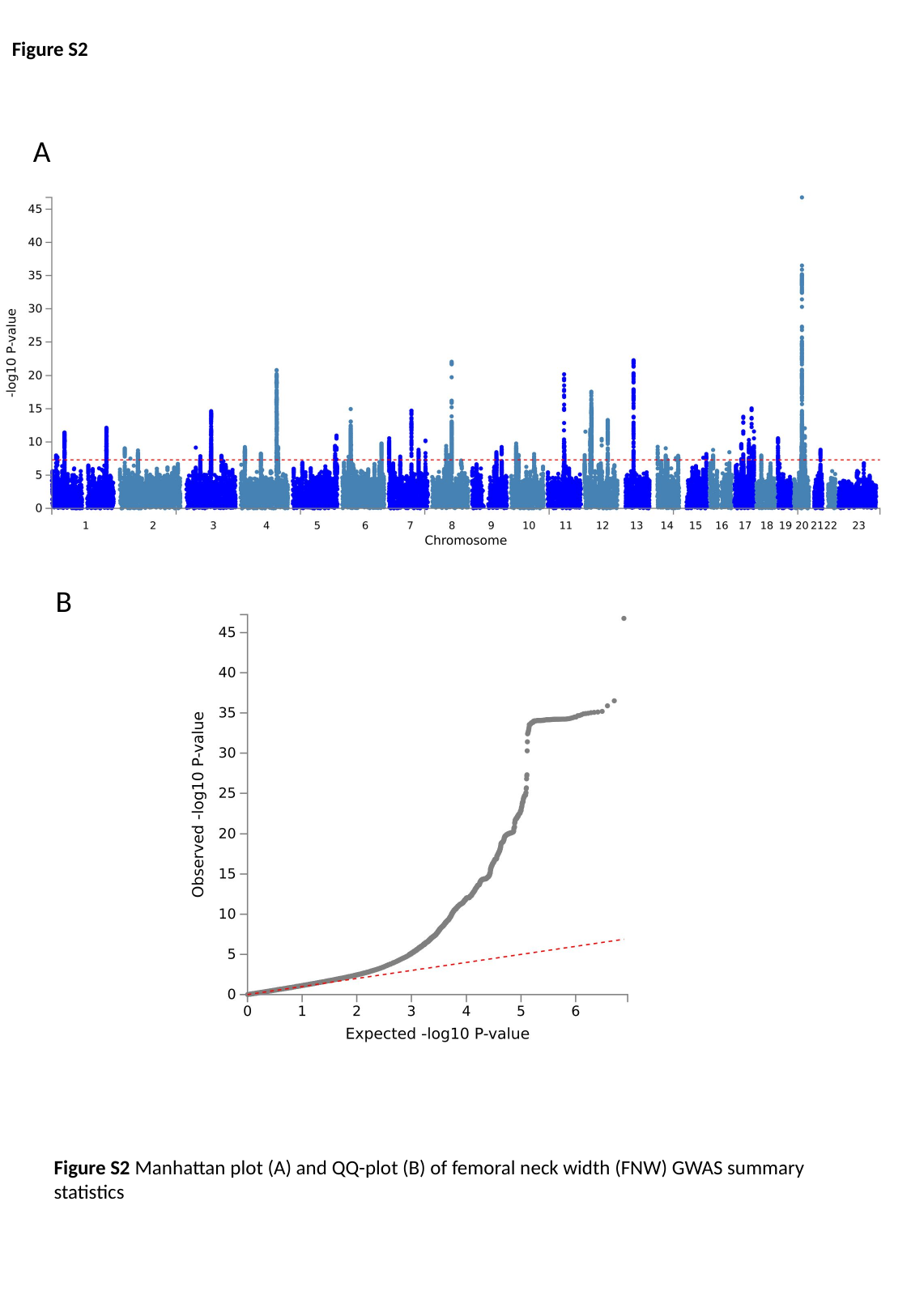

Figure S2
A
B
Figure S2 Manhattan plot (A) and QQ-plot (B) of femoral neck width (FNW) GWAS summary statistics

## Slide 3
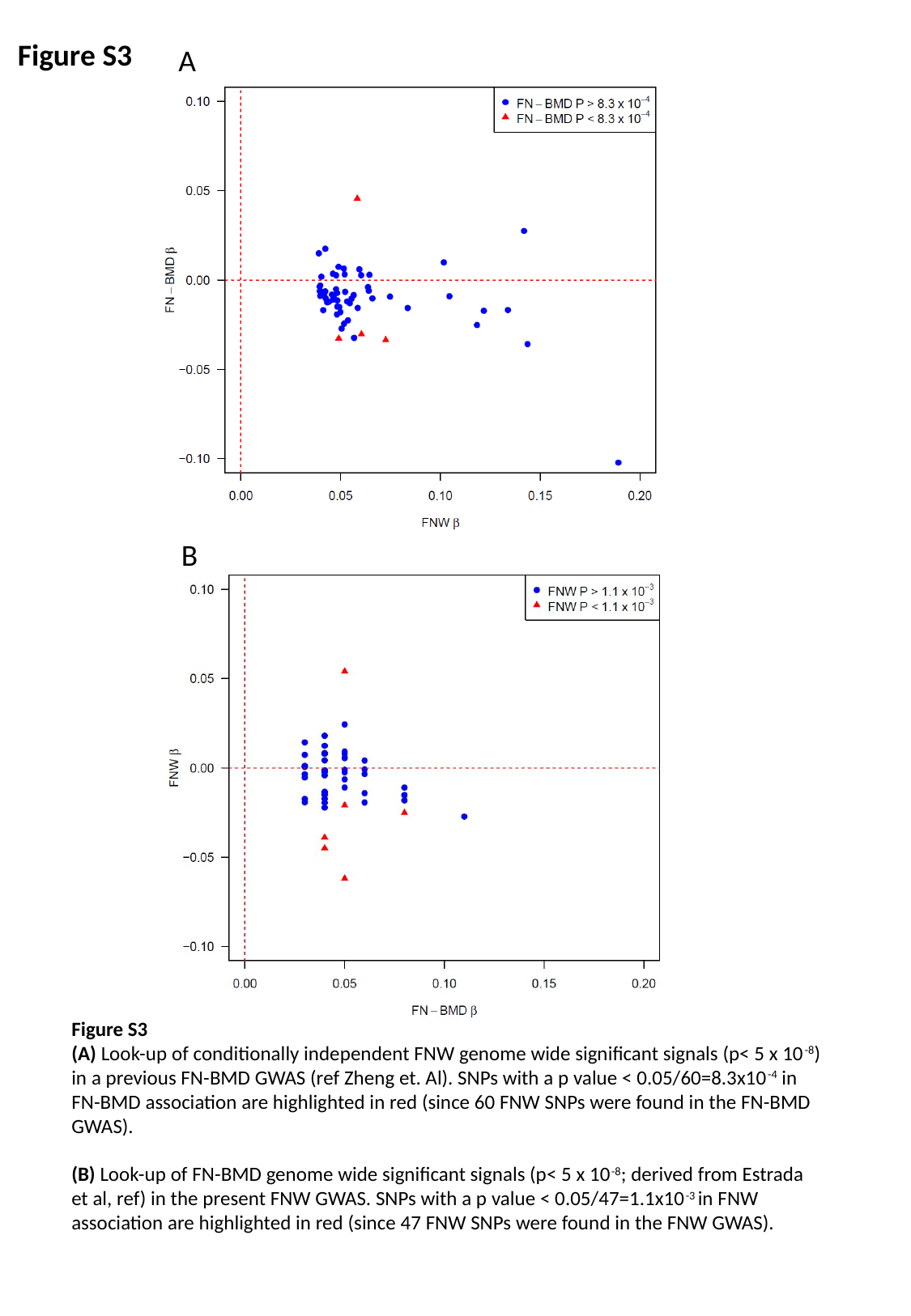

Figure S3
A
B
Figure S3
(A) Look-up of conditionally independent FNW genome wide significant signals (p< 5 x 10^-8^) in a previous FN-BMD GWAS (ref Zheng et. Al). SNPs with a p value < 0.05/60=8.3x10^-4^ in FN-BMD association are highlighted in red (since 60 FNW SNPs were found in the FN-BMD GWAS).
(B) Look-up of FN-BMD genome wide significant signals (p< 5 x 10^-8^; derived from Estrada et al, ref) in the present FNW GWAS. SNPs with a p value < 0.05/47=1.1x10^-3^ in FNW association are highlighted in red (since 47 FNW SNPs were found in the FNW GWAS).

## Slide 4
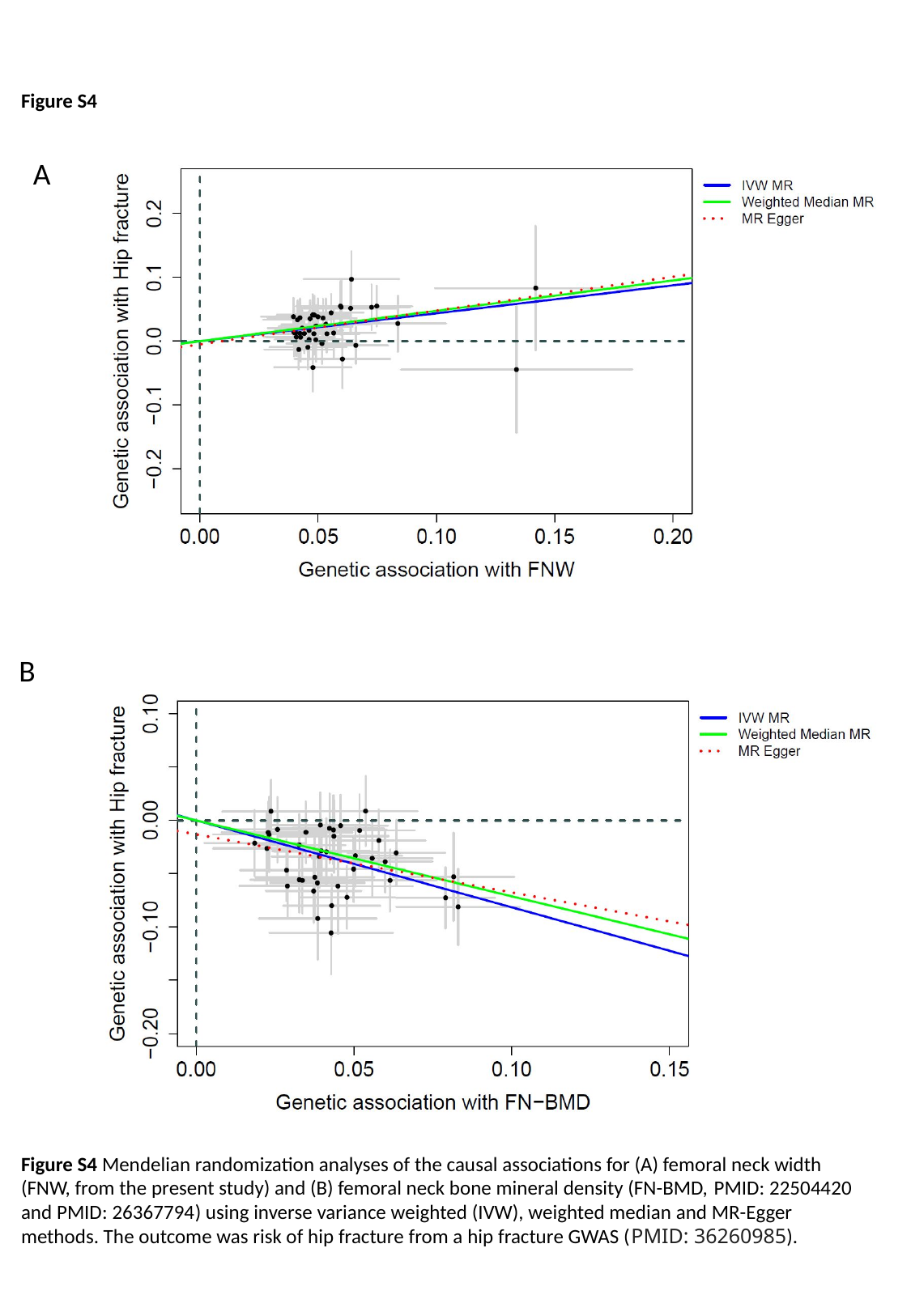

Figure S4
A
B
Figure S4 Mendelian randomization analyses of the causal associations for (A) femoral neck width (FNW, from the present study) and (B) femoral neck bone mineral density (FN-BMD, PMID: 22504420 and PMID: 26367794) using inverse variance weighted (IVW), weighted median and MR-Egger methods. The outcome was risk of hip fracture from a hip fracture GWAS (PMID: 36260985).

## Slide 5
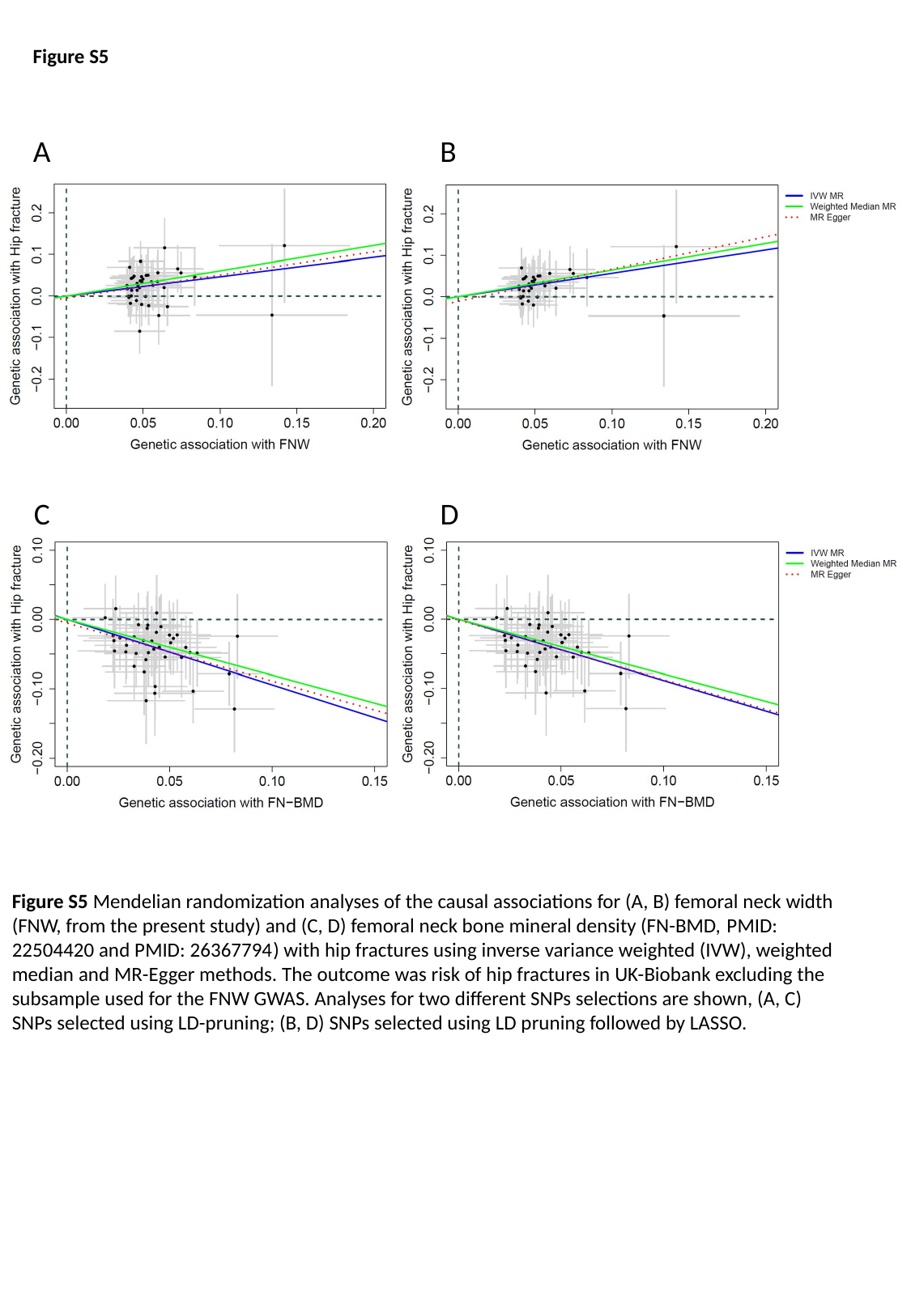

Figure S5
A
B
C
D
Figure S5 Mendelian randomization analyses of the causal associations for (A, B) femoral neck width (FNW, from the present study) and (C, D) femoral neck bone mineral density (FN-BMD, PMID: 22504420 and PMID: 26367794) with hip fractures using inverse variance weighted (IVW), weighted median and MR-Egger methods. The outcome was risk of hip fractures in UK-Biobank excluding the subsample used for the FNW GWAS. Analyses for two different SNPs selections are shown, (A, C) SNPs selected using LD-pruning; (B, D) SNPs selected using LD pruning followed by LASSO.

## Slide 6
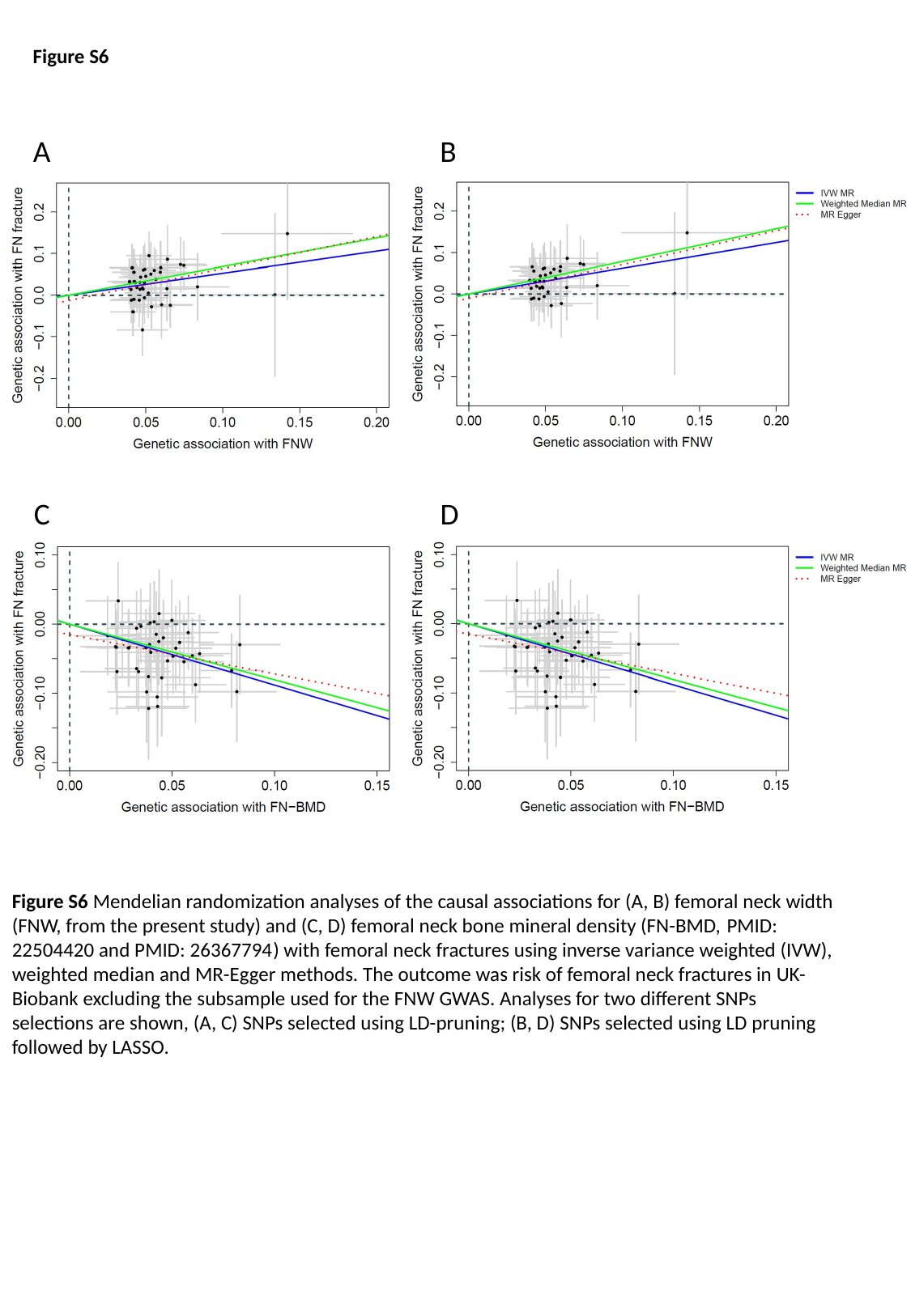

Figure S6
A
B
C
D
Figure S6 Mendelian randomization analyses of the causal associations for (A, B) femoral neck width (FNW, from the present study) and (C, D) femoral neck bone mineral density (FN-BMD, PMID: 22504420 and PMID: 26367794) with femoral neck fractures using inverse variance weighted (IVW), weighted median and MR-Egger methods. The outcome was risk of femoral neck fractures in UK-Biobank excluding the subsample used for the FNW GWAS. Analyses for two different SNPs selections are shown, (A, C) SNPs selected using LD-pruning; (B, D) SNPs selected using LD pruning followed by LASSO.

## Slide 7
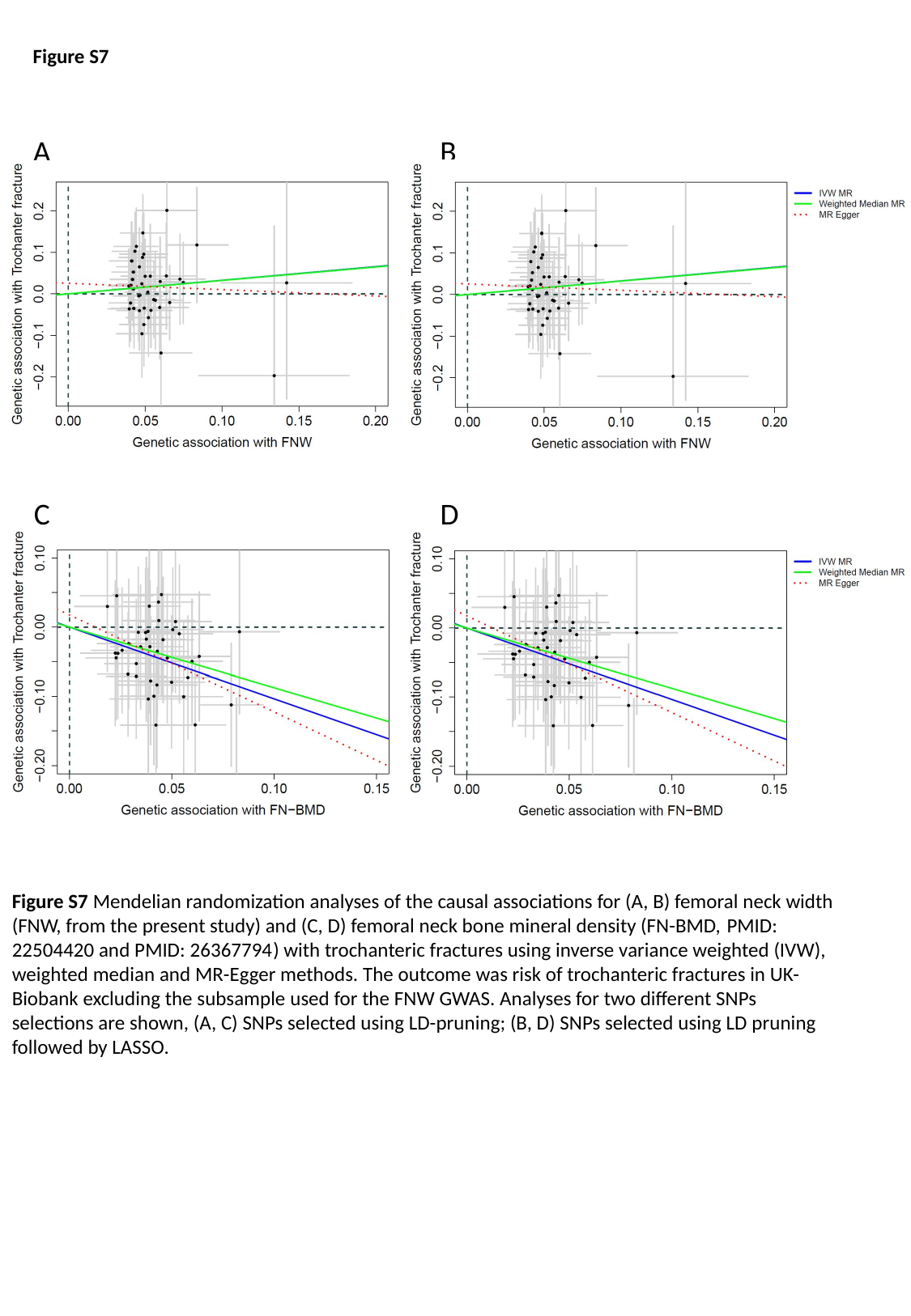

Figure S7
A
B
C
D
Figure S7 Mendelian randomization analyses of the causal associations for (A, B) femoral neck width (FNW, from the present study) and (C, D) femoral neck bone mineral density (FN-BMD, PMID: 22504420 and PMID: 26367794) with trochanteric fractures using inverse variance weighted (IVW), weighted median and MR-Egger methods. The outcome was risk of trochanteric fractures in UK-Biobank excluding the subsample used for the FNW GWAS. Analyses for two different SNPs selections are shown, (A, C) SNPs selected using LD-pruning; (B, D) SNPs selected using LD pruning followed by LASSO.

## Slide 8
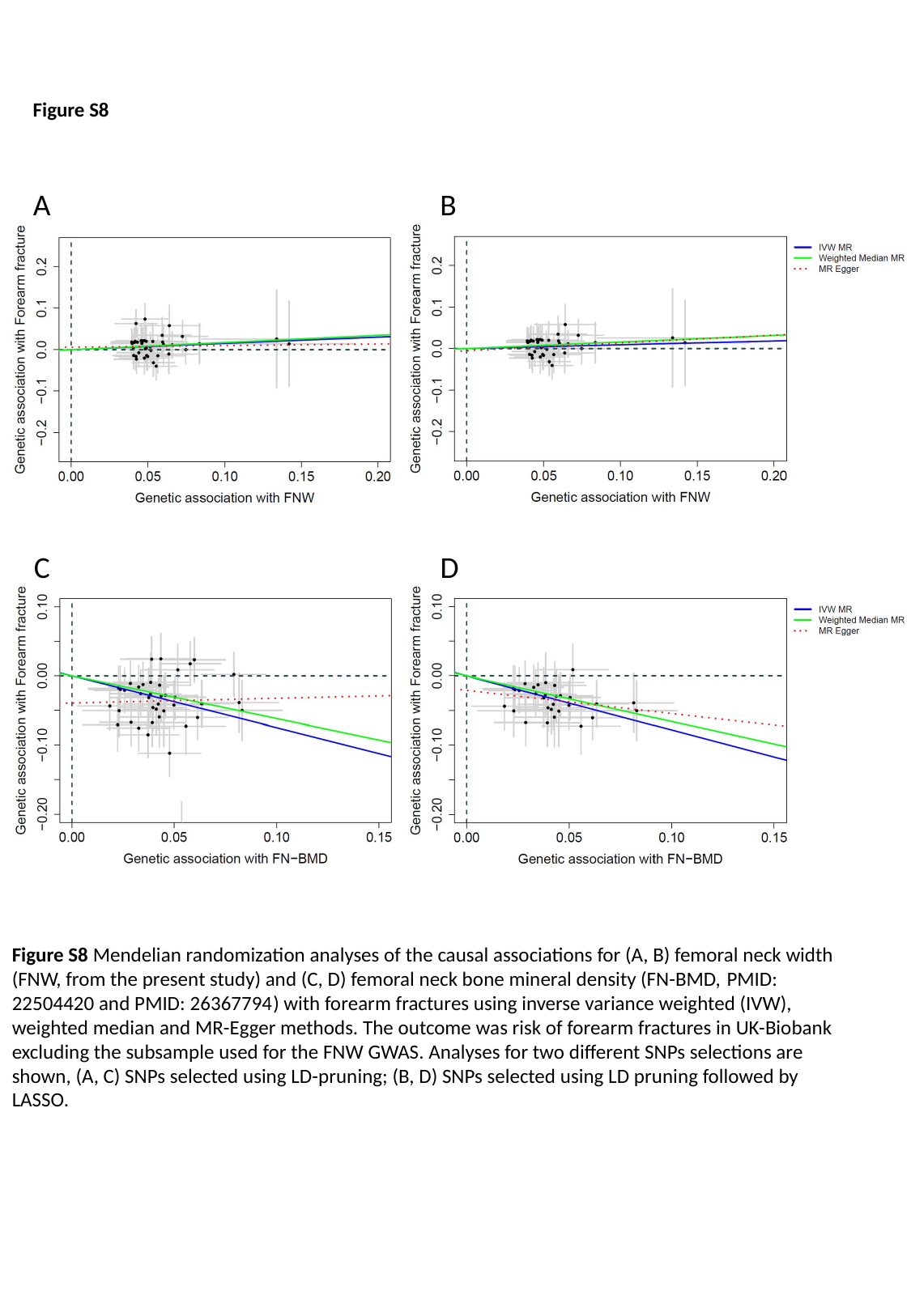

Figure S8
A
B
C
D
Figure S8 Mendelian randomization analyses of the causal associations for (A, B) femoral neck width (FNW, from the present study) and (C, D) femoral neck bone mineral density (FN-BMD, PMID: 22504420 and PMID: 26367794) with forearm fractures using inverse variance weighted (IVW), weighted median and MR-Egger methods. The outcome was risk of forearm fractures in UK-Biobank excluding the subsample used for the FNW GWAS. Analyses for two different SNPs selections are shown, (A, C) SNPs selected using LD-pruning; (B, D) SNPs selected using LD pruning followed by LASSO.
