# Supplementary material for: Femoral neck width genetic risk score is a novel independent risk factor for hip fractures
Source: J Bone Miner Res. 2024 Jan 12;39(3):241–51. doi: 10.1093/jbmr/zjae002 (PMC11240160; doi:10.1093/jbmr/zjae002)
Supplement: Supplementary_Methods_zjae002 [file Supplementary_Methods_zjae002.docx]

**SUPPLEMENTARY METHODS**

**Preparation and Quality control of genetic data in UKB**

Samples were genotyped using two genotyping arrays; Applied Biosystems UK BiLEVE Axiom Array by Affymetrix (49,950 participants) and Applied Biosystems UK Biobank Axiom Array (438,427 participants). Data were imputed using the HRC reference panel, and the merged UK10K and 1000 Genomes phase 3 reference panels in IMPUTE4. A subset of European individuals from UKB with DXA analyses available was used for FNW GWAS. Ancestry assignment of UKB participants was performed as follows: the UKB sample was projected onto the first 20 principal components estimated from the 1000 Genomes Phase 3 (1000G) project (where ancestry was known) using GCTA version 1.93.2. Projections used a curated set of 38,512 LD-pruned HapMap 3 Release 3 (HM3) REF bi-allelic SNPs that were shared between the 1000G and UKB genotyped datasets (i.e. MAF > 1%, minor allele count > 5, genotyping call rate > 95%, Hardy-Weinberg P > 1×10^-6^, and regions of extensive LD removed). Uniform Manifold Approximation and Projection for Dimension Reduction (UMAP) was used in conjunction with the first 20 principal components to cluster 486,445 individuals using the following parameters: min_dist=0.0001, n_components=3, n_neighbors=45, random_state=10293082. UKB participants that clustered together with the 1000G European sub-populations were manually identified by visual inspection (N=461,920) and used for downstream genetic analyses.
